# Ramipril inhibits AGE-RAGE-induced matrix metalloproteinase-2 activation in experimental diabetic nephropathy

**DOI:** 10.1186/1758-5996-6-86

**Published:** 2014-08-13

**Authors:** Kei Fukami, Sho-ichi Yamagishi, Melinda T Coughlan, Brooke E Harcourt, Phillip Kantharidis, Vicki Thallas-Bonke, Seiya Okuda, Mark E Cooper, Josephine M Forbes

**Affiliations:** Division of Nephrology, Department of Medicine, Kurume University School of Medicine, 67 Asahi-machi, Kurume, Fukuoka, 830-0011 Japan; Department of Pathophysiology and Therapeutics of Diabetic Vascular Complications, Kurume University School of Medicine, Kurume, Japan; Diabetes Division, Baker IDI Heart and Diabetes Institute, Melbourne, Australia; Department of Medicine, Central Clinical School, Monash University, Melbourne, Australia; Department of Glycation and Diabetic Complications, Mater Medical Research Institute, Brisbane, Australia

**Keywords:** Advanced glycation end products, Diabetic nephropathy, Renin-angiotensin system, MMP-2, RAGE, NF-κB

## Abstract

**Background:**

Advanced glycation end products (AGE)-receptor for AGE (RAGE) axis and renin-angiotensin system (RAS) play a role in diabetic nephropathy (DN). Matrix metalloproteinase-2 (MMP-2) activation also contributes to DN. However, the pathological interaction among AGE-RAGE, RAS and MMP-2 in DN remains unknown. We examined here the involvement of AGE and RAS in MMP-2 activation in streptozotocin (STZ)-induced diabetic rats and in AGE-exposed rat renal proximal tubular cells (RPTCs).

**Methods:**

Experimental diabetes was induced in 6-week-old male Sprague–Dawley (SD) rats by intravenous injection of STZ. Diabetic rats received ramipril (3 mg/kg body weight/day) or vehicle for 32 weeks. AGE-modified rat serum albumin (AGE-RSA) or RSA was intraperitoneally administrated to 6-week-old male SD rats for 16 weeks. RPTCs were stimulated with 100 μg/ml AGE-modified bovine serum albumin (AGE-BSA) or BSA in the presence or absence of 10^-7^ M ramiprilat, an inhibitor of angiotensin-converting enzyme or 100 nM BAY11-7082, an IκB-α phosphorylation inhibitor.

**Results:**

AGE and RAGE expression levels and MMP-2 activity in the tubules of diabetic rats was significantly increased in association with increased albuminuria, all of which were blocked by ramipril. AGE infusion induced tubular MMP-2 activation and RAGE gene expression in SD rats. Ramiprilat or BAY11-7082 inhibited the AGE-induced MMP-2 activation or reactive oxygen species generation in RPTCs. Angiotensin II increased MMP-2 gene expression in RPTCs, which was blocked by BAY11-7082.

**Conclusions:**

Our present study suggests the involvement of AGE-RAGE-induced, RAS-mediated MMP-2 activation in experimental DN. Blockade of AGE-RAGE axis by ramipril may protect against DN partly *via* suppression of MMP-2.

## Introduction

Diabetic nephropathy (DN) is a leading cause of end-stage renal disease, which could account for disability and high mortality rate in patients with diabetes [1, 2]. The development and progression of DN are characterized by glomerular hypertrophy and inflammatory cell infiltration, followed by extracellular matrix (ECM) accumulation in mesangial area and an increased albumin excretion rate [3]. Diabetic nephropathy ultimately progresses glomerular sclerosis associated with renal dysfunction [4]. However, it has recently been recognized that changes within tubulointerstitium are more important than glomerulopathy in terms of renal dysfunction in DN [5, 6].

Reducing sugars can react non-enzymatically with the amino groups of proteins to initiate a complex series of rearrangements and dehydrations, and then to produce a class of irreversibly cross-linked moieties termed advanced glycation end products (AGE) [7–9]. The formation and accumulation of AGE in various tissues have been shown to progress at an accelerated rate under hyperglycemic conditions [10–12]. There is accumulating evidence that AGE and receptor for AGE (RAGE) interaction induces oxidative stress generation and subsequently evokes inflammatory reactions, thereby causing progressive alteration in renal architecture and loss of renal function in diabetes [13–15].

Matrix metalloproteinases (MMPs) are a family of zinc-dependent endopeptidases comprising more than 20 members that can degrade numerous types of ECM components [16]. Among various MMPs, MMP-2 has attracted great attention because it can degrade type-I and -IV collagen and laminin, major components of tubular basement membrane and interstitium [17, 18]. Recently, we have found that serum MMP-2 levels are one of the independent determinants of proteinuria in patients with chronic kidney disease [19]. Further, plasma MMP-2 levels and its activity are significantly higher in type-1 diabetic patients compared with control subjects [20], and urinary MMP-2 values are positively associated with renal hyperfiltration and albuminuria in these diabetic patients as well [20]. In addition, in animal models, MMP-2 has been shown to induce renal tubular cell epithelial-mesenchymal transformation, which could cause tubulointerstitial fibrosis in diabetic nephropathy [21, 22]. These observations suggest the involvement of MMP-2 activation in albuminuria and tubulointerstitial injury of diabetic nephropathy [23].

There is accumulating evidence to show the active participation of renin-angiotensin system (RAS) in DN as well [24]. Indeed, inhibition of RAS by angiotensin-converting enzyme (ACE) inhibitor or angiotensin II (Ang II) type-1 receptor (AT1R) blocker has been shown to suppress the development and progression of nephropathy in both type-1 and type-2 diabetic subjects [25, 26]. Further, losartan, an AT1R blocker, improves renal outcome in patients with type-2 diabetes [27]. Moreover, we have previously found that RAS blockers could inhibit the AGE-elicited mesangial cell hypertrophy, proximal tubular cell injury, and podocyte DNA damage and detachment *in vitro* [28–30]. However, the involvement of AGE and RAS in MMP-2 activation in DN remains unknown. Therefore, we first examined the effects of ramipril, an inhibitor of ACE on MMP-2 activity, AGE and RAGE expression in renal tubules of streptozotocin (STZ)-induced diabetic rats. Then we investigated whether AGE injection could stimulate RAGE gene expression and MMP-2 activity in tublules of normal non-diabetic rats. We further studied the effects of ramiprilat, an active metabolite of ramipril, on MMP-2 activity and reactive oxygen species (ROS) generation in AGE-exposed rat renal proximal tubular cells (RPTCs).

## Methods

### Experimental animal models

Experimental diabetes was induced in 6-week-old male Sprague–Dawley (SD) rats (200–250 g) by intravenous injection of STZ (50 mg/kg body weight) in sodium citrate buffer pH 4.5, following an overnight fast [31]. Rats with plasma glucose concentrations in excess of 15 mmol/L were included in this study. Vehicle-injected control (Ctrl) animals (n = 15) were followed concurrently. Diabetic rats were randomized into two groups and followed for 32 weeks; one group (n = 15) received a vehicle, and the other an ACE inhibitor, ramipril (1 mg/kg body weight/day in drinking water; generously provided by Sanofi, Bridgewater, NJ) for 32 weeks (n = 16) [32]. Two units of ultralente insulin (Ultratard HM, Novo Industries, Bagsvaerd, Denmark) were administrated daily to diabetic animals to prevent ketoacidosis and avoid death. In addition, non-diabetic normal male SD rats (6-week-old) were infused intraperitoneally with AGE-modified rat serum albumin (AGE-RSA) (n = 10) or RSA (n = 9) at a dose of 20 mg/kg body weight/day for 16 weeks by an osmotic pump (Alzet osmotic pumps, model 1004, Cupertino, CA, USA).

Systolic blood pressure (SBP) was measured by tail-cuff plethysmography as described previously [33]. Glomerular filtration rate (GFR) was evaluated by ^99^Tc-DTPA, and urinary albumin excretion (UAE) levels by an enzyme-linked immunosorbent assay (ELISA) kit (Bethyl Laboratories, Montgomery, TX, USA). Other clinical valuables were measured as described previously [31]. All animal procedures were in accordance with guidelines set by the Baker IDI Heart and Diabetes Institute Ethics Committee and the National Health and Medical Research Council of Australia.

### Preparation of AGE-RSA and AGE-modified bovine serum albumin (AGE-BSA)

AGE-RSA and AGE-BSA were prepared by incubating RSA or BSA (Fraction V, Sigma Chemical Co, St. Louis, MO, USA) with 0.5 M D-glucose in PBS at 37°C for 3 months as previously described [34]. After sterilization using 0.2 μm micropore filters, unincorporated glucose was removed by dialysis against phosphate buffer saline (PBS) at 4°C for 48 hr. Samples were passed through Detoxigel column (Pierce Biotechnology Inc., Rockford, IL, USA) in order to remove endotoxin. Preparations were tested for endotoxin using Limulus Amebocyte Lysate validity testing (AMS Laboratories, Sydney, Australia); no endotoxin was detected. Finally, the solution was filtered through 0.2 μm micropore filter in sterile conditions, and percentage of lysine modifications and carboxymethyllysine (CML) moieties were determined by Selective Ion Monitoring Gas chromatography–mass spectrometry as previously described [35]. Control non-glycated RSA or BSA was incubated in the same conditions except for the absence of glucose.

### Isolation of renal tubules from kidneys

The cortical tissue was minced and gently pushed through a 250 μm steinless steel mesh with 0.9% sodium chloride solution, and sieved stepwise through 125 μm and 75 μm meshes. The tubules were collected on the 125 μm mesh as described previously [36]. Tubular structures were confirmed by light microscopy. After centrifugation, the tubules were re-suspended in the lysis buffer (50 mM Tris–HCl, 150 mM NaCl, 0.02% sodium azide, 0.1% SDS, 1% Nonidet P-40, 0.5% sodium deoxycholate) containing a complete protease inhibitor cocktail (Roche Molecular Biochemicals, Mannheim, Germany) without ethylenediaminetetraacetic acid (EDTA). Then the samples were sonicated and the protein concentration was measured using a BCA protein assay kit (Pierce Biotechnology Inc.).

### Gelatin zymography

Twenty μg protein isolated from renal tubules or cell culture media were separated by 8% sodium dodecyl sulfate-polyacrylamide gel electrophoresis (SDS-PAGE) containing 1 mg/ml gelatin (Labchem, Auburn, NSW, Australia), and active MMP-2 levels were evaluated by zymography as previously described [14].

### Measurement of AGE levels in tubules

AGE levels in the isolated tubules were measured with CML ELISA system as previously described [37]. In brief, 1 μg of samples diluted in 50 mM carbonate buffer (pH 9.6) (1:800) or standards were added to a microtitre plate (Nunc-Immuno MaxiSorp, Nunc, Kamstrup, Roskilde, Denmark) and incubated overnight at 4°C. After washing three times with PBS (pH 7.4) containing 0.1% Tween-20, wells were blocked for 1 hr with PBS containing 1% BSA, and then 100 μl of a rabbit polyclonal anti-CML antibody (5 μg/ml) was added [38]. After 1 hr incubation and washing, 100 μl of 0.2 μg/ml goat-anti-rabbit IgG biotinylated antibody (Dako Corporation, Carpinteria, CA, USA) was added to each well. After 1 hr of shaking, wells were washed and 100 μl of streptavidin horseradish peroxidase (Dako Corporation) was added for 30 min. The wells were washed and 100 μl of Tetramethylbenzidine (Sigma Chemical Co.) was added, the reaction being terminated after 15 min using 100 μl 1.8 M H_2_SO_4_. The absorbance was quantitated using a microtitre plate reader at 450 nm (EMax, Molecular Devices Corporation, Sunnyvale, CA, USA).

### Western blot analysis

Ten μg protein isolated from renal tubules was separated by 10% SDS-PAGE electrophoresis, and then transferred to a polyvinylidene fluoride membrane (Hybond P; Amersham, Buckinghamshire, UK). Membranes were incubated with mouse anti-RAGE (1:1000) or rabbit anti-α-tubulin (1:1000) antibodies overnight, and then the secondary antibody horseradish peroxidase-conjugated mouse or rabbit IgG for 1 hr. Bound antibodies were detected by reaction with an enhanced chemiluminescence kit (Pierce Biotechnology Inc.). All antibodies were obtained from Chemicon (Santa Cruz, SA, USA).

### Quantitative real-time reverse-transcription polymerase chain reaction (RT-PCR)

About 4 μg of total RNA extracted from each kidney cortex or RPTCs were used to synthesize cDNA with the SuperScript First-Strand Synthesis System for RT-PCR (Invitrogen, Carlsband, CA, USA) as previously described [35]. Quantitative real-time RT-PCR was performed using Assay-on-Demand and TaqMan 5 fluorogenic nuclease chemistry (Applied Biosystems, Foster City, CA, USA) according to the supplier’s recommendation. The forward, reverse primers and specific probes for rat RAGE and MMP-2 genes were 5′-TCCTGGTGGGACCGTGAC-3′, 5′-GGGTGTGCCATCTTTTATCCA-3′, and FAM5′-TGTGCCATCTCTGC-3′-MGB, and 5′-GCCCCTATCTACACCTACACCAA-3′, 5′-TGGATCCCCTTGATGTCATCA-3′, and FAM-5′-AACTTCCGATTATCCC-3′-MGB, respectively. TaqMan Ribosomal RNA Control Reagents (18S) was used as an endogenous control (Applied Biosystems).

### Isolation and characterization of RPTCs

RPTCs were isolated from rat kidney as previously described [39]. In brief, kidneys were removed from male SD rats (200–250 g), and renal cortex was minced and centrifuged at 250 *g*, 4°C for 5 min. Final pellets were digested in Dulbecco’s Modified Eagle’s Medium/Ham’s F12 (1:1) containing 1 mg/mL collagenase (Type-2, Worthington Biochemical Corporation, Lakewood, NJ, USA) for 30 min at 37°C with constant agitation. This suspension was filtered through a 75 μm pore size metal sieve to remove glomeruli, and re-suspended in a 50% Percoll solution (Sigma-Aldrich Chemie GmbH, Buchs, Switzerland) followed by centrifugation at 26,500 *g* at 4°C for 30 min. The lowest band was retrieved as it is enriched for proximal tubule fragments at a purity of greater than 98% as described previously [40]. RPTCs were characterized as cobble stone-like appearance and immunocytochemical characteristics with positive staining for cytokeratin and P. vulgaris lectin in the absence of CD90.1 (thy1.1) using confocal microscopy. Treatments with AGE-BSA, Ang II (Sigma Chemical Co.), an inhibitor of ACE, ramiprilat, or an inhibitor of IκB-α phosphorylation, BAY11-7082 ((E)-3-(4-methylphenylsulfonyl)-2-propenenitrile) (Biomol international, Lp, USA) [41] were carried out in Minimum Essential Medium containing D-valine instead of L-valine without fetal calf serum in humidified 5% CO_2_/95% air atmosphere at 37°C.

### Intracellular ROS generation

RPTCs were treated with 100 μg/ml AGE-BSA or BSA in the presence or absence of 10^-7^ M ramiprilat for 24 hr, and then intracellular ROS generation was measured using the fluorescent probe 5,6-chloromethyl- 2′,7′-dichlorohydrofluorescein diacetate (Molecular Probes, Eugene, OR, USA) as described previously [28].

### Statistical analysis

Results are expressed as mean ± standard error. Data for albuminuria were not normally distributed, therefore analyzed as logarithmic transformation. One-way ANOVA followed by the Tukey-test or unpaired t-test was performed for statistical comparisons; p < 0.05 was considered significant. All statistical analyses were performed with SPSS system (Ver. 20, SPSS, Chicago, IL, USA).

## Results

### Characteristics of animals

Compared with non-diabetic Ctrl rats, plasma glucose, glycated hemoglobin (HbA1c), SBP, kidney-to-body weight ratio, GFR and UAE levels were significantly higher in diabetic rats (p < 0.001) (Table 1). SBP was partially, but not significantly, decreased after ramipril treatment (Table 1). Furthermore, although plasma glucose, HbA1c or GFR levels were not affected by the treatment with ramipril, it significantly reduced the UAE levels in diabetic rats (p < 0.001) (Table 1).

**Table 1:** **Characteristics of animals**

	Ctrl	DM	DM + ramipril	RSA	AGE-RSA
Number	15	15	16	9	10
Plasma glucose (mmol/L)	6.9 ± 095	33.2 ± 2.7*	33.1 ± 3.5*	7.9 ± 1.0	7.5 ± 0.5
%HbA1c (%)	5.5 ± 0.7	18.3 ± 2.6*	19.3 ± 2.7*	4.1 ± 1.5	3.9 ± 0.8
Systolic BP (mmHg)	114 ± 9	134 ± 13*	122 ± 8^#^	121 ± 4	118 ± 7
KW/BW ratio	5.3 ± 0.6	10.9 ± 1.5*	11.3 ± 1.1*	5.4 ± 0.3	5.4 ± 0.4
eGFR (ml/min)	6.7 ± 1.1	11.2 ± 1.4*	12.0 ± 1.7*	4.3 ± 1.0	4.4 ± 0.6
UAE (mg/24 h)	5.6 ± 4.3	53.9 ± 51.8*	13.0 ± 6.8^$^	1.0 ± 0.4	1.3 ± 0.5

Data are mean ± SEM. *p < 0.001 vs Ctrl, ^#^p < 0.05 vs Ctrl, ^$^p < 0.001 vs DM.

*Abbreviation: Ctrl* control, *DM* diabetes mellitus, *RSA* rat serum albumin, *AGE-RSA* advanced glycation end product-modified RSA, *HbA1c* glycated hemoglobin, *BP* blood pressure, *KW/BW ratio* kidney-to-body weight ratio, *GFR* glomerular filtration rate, *UAE* urinary albumin excretion.

Compared with RSA treatment, AGE-RSA injection tended to increase UAE levels, but the effects were modest, not significant (p = 0.13) (Table 1). In addition, AGE-RSA injection did not affect any clinical parameters in SD rats compared with RSA infusion.

### Ramipril decreased MMP-2 activity, AGE and RAGE expression in the tubules of diabetic rats

**Figure 1:**
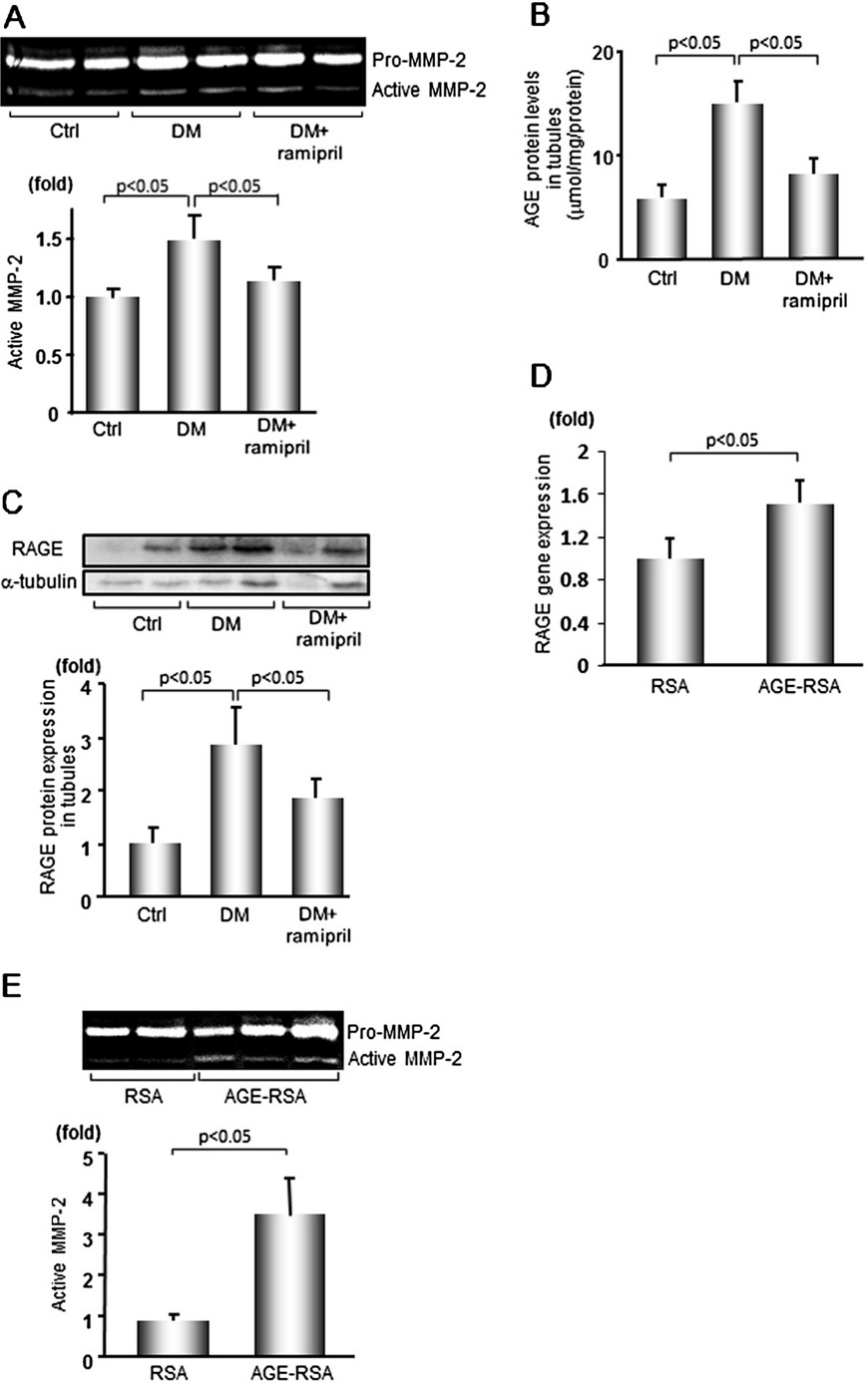
**MMP-2 activity, AGE accumulation and RAGE expression in the renal tubules of diabetic or AGE-RSA-infused rats. (A)** MMP-2 activity in 32-week Ctrl or diabetic renal tubules with or without ramipril treatment was determined by gelatin zymography (*n* = 4). **(B)** AGE protein levels in 32-week Ctrl or diabetic renal tubules with or without ramipril treatment were evaluated by enzyme-linked immunosorbent assay for carboxymethyllisine (*n* = 7). **(C)** RAGE protein expression in 32-week Ctrl or diabetic renal tubules with or without ramipril treatment was determined by western blot. Data were normalized by the intensity of α-tubulin bands. (*n* = 4). **(D)** RAGE gene expression in total kidney cortex of AGE-RSA- or RSA-infused rats was evaluated by quantitative real-time RT-PCR (n = 7). **(E)** MMP-2 activity in the tubules of AGE-RSA- or RSA-infused rats was determined by gelatin zymography (n = 4). Data are shown as mean ± SEM.

As shown in Figure 1A-C, compared with Ctrl rats, MMP-2 activity, AGE and RAGE expression levels in the tubules of diabetic rats was significantly increased, all of which were inhibited by the treatment with ramipril.

### AGE-RSA infusion increased tubular MMP-2 activity and RAGE gene expression in SD rats

We next investigated whether AGE-RSA could directly induce MMP-2 activation in tubules. We evaluated renal rather than tubular RAGE gene expression in the present study because (1) tubules and interstitium make up approximately 80-90% of the renal volume and (2) isolation of tubules from the kidney cortex might affect mRNA stability and expression level [42]. As shown in Figure 1D and E, compared with RSA-infused rats, renal RAGE gene expression and tubular MMP-2 activity were significantly increased in AGE-RSA-infused rats.

### Ramiprilat suppressed the AGE-elicited MMP-2 activation and ROS generation in RPTCs

**Figure 2:**
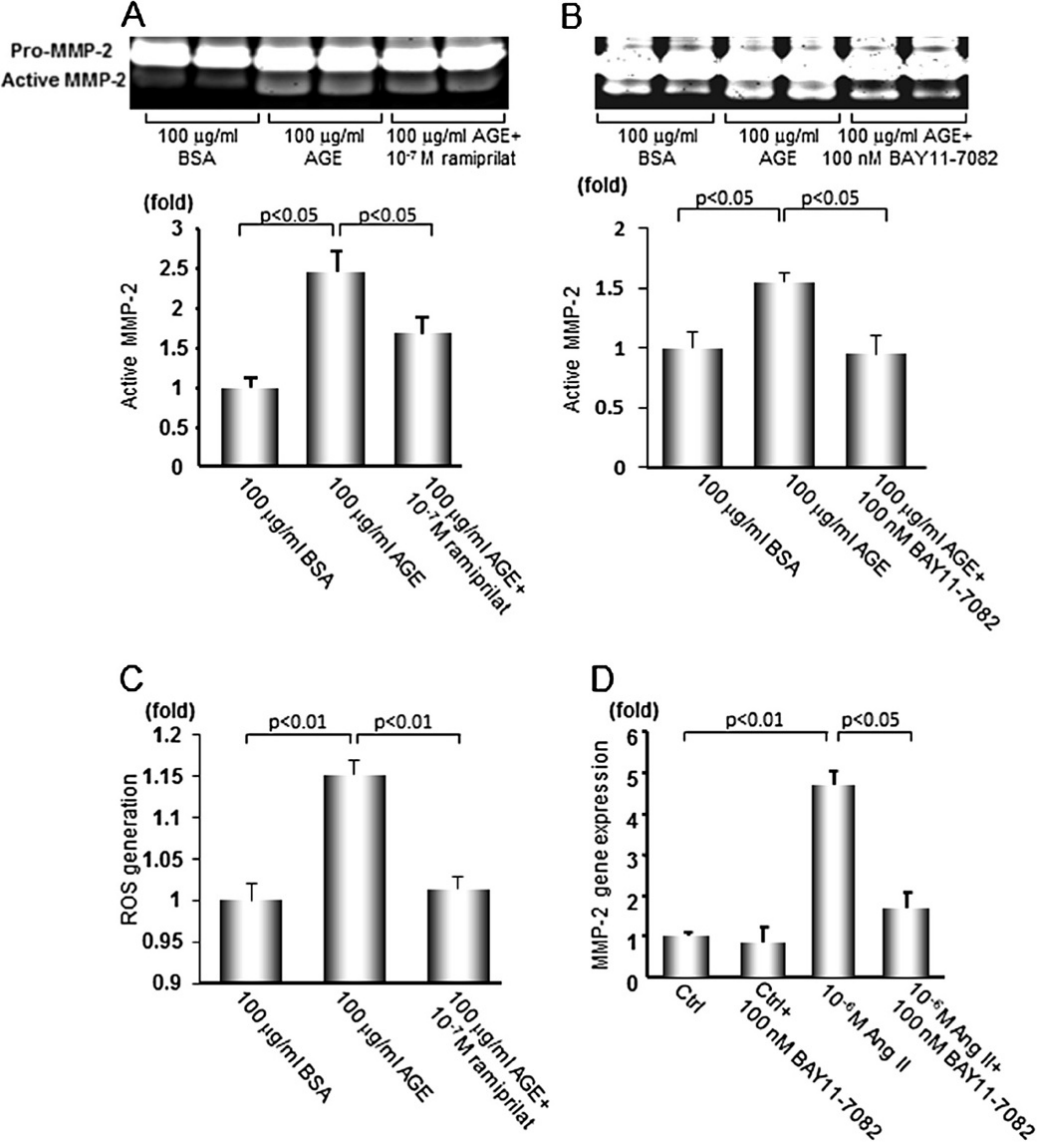
**MMP-2 activity and gene expression, and intracellular ROS generation in RPTCs. (A, B)** RPTCs were stimulated with 100 μg/ml AGE-BSA or non-glycated BSA with or without 10^-7^ M ramiprilat or 100 nM BAY11-7082 for 48 hr, and MMP-2 activity in the supernatant was determined by gelatin zymography (n = 4). **(C)** Intracellular ROS generation in RPTCs. Cells were stimulated with 100 μg/ml AGE-BSA or BSA with or without 10^-7^ M ramiprilat for 24 hr, then the intracellular ROS generation in RPTCs was evaluated using fluorescent probe 10 μM CM-H_2_DCFDA. **(D)** Effects of Ang II on MMP-2 gene expression in RPTCs. The cells were stimulated with 10^-6^ M Ang II with or without 100 nM BAY11-7082 for 24 hr and MMP-2 gene expression was evaluated by quantitative real-time RT-PCR (n = 6). Data are shown as mean ± SEM.

We examined whether and how AGE could induce MMP-2 activation *in vitro*. Since AGE or Ang II exert various biological effects *via* activation of redox-sensitive transcription factor NF-κB, we studied the effects of BAY11-7082, an inhibitor of NF-κB activation on AGE- or Ang II-induced MMP-2 gene expression and activity in PRTCs. Treatment with 100 μg/ml AGE-BSA significantly increased active MMP-2 production by RPTCs, which was completely prevented by 10^-7^ M ramiprilat or 100 nM BAY11-7082 (Figure 2A and B). Ramiprilat completely blocked the AGE-induced ROS generation in RPTCs, whereas 10^-6^ M Ang II significantly increased MMP-2 gene expression, which was also inhibited by BAY11-7082 (Figure 2C and D).

## Discussion

In the present study, we demonstrated that (1) MMP-2 activity, AGE and RAGE expression levels were significantly increased in renal tubules of diabetic rats, all of which were blocked by the treatment with ramipril; (2) UAE, a prognostic marker of DN, was increased in diabetic rats, which was also inhibited by ramipril; (3) AGE injection significantly increased renal RAGE gene expression and MMP-2 activity in the tubules of normal SD rats; (4) AGE treatment enhanced MMP-2 activity and ROS generation in RPTCs, which were also suppressed by ramiprilat; and (5) inhibition of NF-κB activation by BAY11-7082 blocked the MMP-2 activity or gene expression in AGE- or Ang II-exposed RPTCs, respectively.

**Figure 3:**
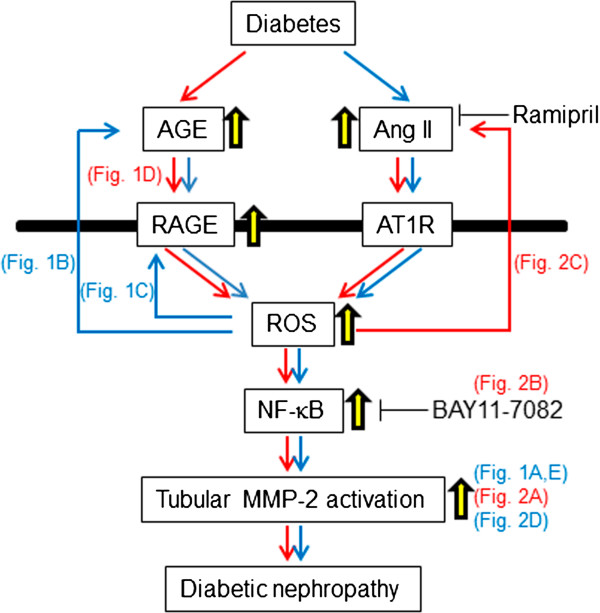
**Possible mechanisms involved in diabetes-elicited tubular MMP-2 activation** ***via*** **AGE-RAGE-RAS axis in diabetic nephropathy.**

In this study, ramipril decreased the AGE accumulation and RAGE expression in the renal tubules of diabetic rats. We have previously shown that there could exist the crosstalk between AGE-RAGE axis and RAS in the pathogenesis of DN [14]. Indeed, irbesartan, an AT1R blocker, inhibited the AGE-induced apoptotic cell death and inflammatory and fibrotic reactions in human PTCs by reducing ROS generation *via* suppression of RAGE expression [29] (AGE-RAGE → RAS). Furthermore, AGE elicited mesangial cell hypertrophy by inducing Ang II production, which was also blocked by candesartan, other type of AT1R blocker [28] (AGE-RAGE → RAS). In addition, Ang II infusion increased RAGE expression in retinas *via* ROS generation [43] (RAS → AGE-RAGE). Consistent with these findings, treatment with low-dose valsartan, an AT1R antagonist, decreased serum AGE levels in association with reduced oxidative stress generation in type-2 diabetic patients [44] (RAS → AGE-RAGE). Therefore, ramipril could inhibit the AGE-RAGE system by suppression the RAS (Figure 3). Engagement of RAGE with AGE stimulates ROS generation, which could promote the formation and accumulation of AGE and subsequent RAGE expression, thereby making a vicious cycle between RAGE-downstream signaling pathways and AGE formation in a variety of cells [45, 46]. Therefore, it is also conceivable that ramipril could break the vicious cycle between AGE and RAGE downstream pathway by blocking the crosstalk between the RAS and AGE-RAGE *via* inhibition of ROS generation (Figure 3). In our *in vitro* experiment, ramiprilat completely inhibited AGE-elicited ROS generation in RPTCs, which could support our speculation.

In our study, we demonstrated that RAS inhibition by ramipril suppressed diabetes or AGE-induced MMP-2 activation *in vivo* and *vitro*. It has been reported that AGE-RAGE interaction induces MMP-2 expression and activation in several cell lines [47, 48]. The interaction of AGE-modified amyloid β and RAGE has been shown to induce MMP-2 expression and vascular inflammatory stress in brain endothelial cells [47]. MMP-2 activity in gingival extracts from diabetic mice was significantly increased, which was inhibited by the administration with soluble RAGE, acting as a decoy receptor for AGE [48]. Further, increased vascular ROS generation and NF-κB activation also promote MMP-2 activity in rat model of renovascular hypertension [49]. In our present study, AGE-induced MMP-2 activation was inhibited by the blockade of NF-κB, thus suggesting that AGE-RAGE-mediated ROS generation might induce MMP-2 activation *via* NF-κB. In addition, Ang II-induced MMP-2 expression was completely suppressed by the inhibition of NF-κB in RPTCs as well. These observations suggest that AGE-RAGE axis might stimulate Ang II generation, which could evoke the ROS-NF-κB signaling pathway, thereby being implicated in the tubular MMP-2 activation in diabetes.

It has been shown that decreased glomerular MMP-2 activity is associated with increased mesangial ECM accumulation and glomerular sclerosis in DN [50]. However, there is still controversy about the role of MMP-2 in DN. It is reported that the increased MMP-2 activation in tubules was involved in the development and progression of tubulointerstitial injury [51]. Furthermore, we have recently found that serum MMP-2 levels were positively associated with proteinuria and inversely correlated with estimated GFR in patients with chronic kidney disease [19]. These observations further support the concept that ramipril might inhibit AGE-RAGE axis, which could slow down the development and progression of tubulointerstitial injury by reducing albuminuria in DN *via* suppression of MMP-2.

### Limitations

We performed experiments in Figure 2A and B, separately. This is a reason why two gels looked slightly different and the fold increase in active MMP-2 after AGE is lower in Figure 2B in respect to Figure 2A. However, there was no statistically significant difference of the increase of active MMP-2 after AGE treatment between Figure 2A and B experiments (p = 0.11). Although the increase of ROS generation in RPTCs stimulated with AGE versus BSA was modest, we found here that (1) ramipril significantly suppressed the AGE-induced MMP-2 activation and ROS generation in RPTCs and (2) inhibition of NF-κB by BAY11-7082 completely inhibited the increase of active MMP-2 in AGE-exposed cells. These findings suggest the biological significance of modest ROS generation in the AGE-signaling.

## Conclusions

In this study, we found that MMP-2 activity, AGE and RAGE expressions were increased in renal tubules of type 1 diabetic rats, all of which were blocked by the treatment with ramipril. Further, AGE infusion induced tubular MMP-2 activation and RAGE gene expression in rats. Ramiprilat inhibited the AGE-induced MMP-2 activation or reactive oxygen species generation in RPTCs. These findings suggest the involvement of AGE-RAGE-induced, RAS-mediated MMP-2 activation in experimental DN. Blockade of AGE-RAGE axis by ramipril may protect against DN partly *via* suppression of MMP-2.

## Abbreviations

DN: Diabetic nephropathy
ECM: Extracellular matrix
AGE: Advanced glycation end products
RAGE: Receptor for AGE
MMPs: Matrix metalloproteinases
RAS: Renin-angiotensin system
ACE: Angiotensin-converting enzyme
Ang II: Angiotensin II
AT1R: Ang II type-1 receptor
STZ: Streptozotocin
ROS: Reactive oxygen species
rat RPTCs: Renal proximal tubular cells
SD: Sprague–Dawley
Ctrl: Control
SBP: Systolic blood pressure
GFR: Glomerular filtration rate
UAE: Urinary albumin excretion
ELISA: Enzyme-linked immunosorbent assay
AGE-BSA: AGE-modified bovine serum albumin
PBS: Phosphate buffer saline
CML: Carboxymethyllysine
EDTA: Ethylenediaminetetraacetic acid
SDS-PAGE: Sodium dodecyl sulfate-polyacrylamide gel electrophoresis
RT-PCR: Reverse-transcription polymerase chain reaction
HbA1c: Glycated hemoglobin.
